# Supplementary material for: HIVprotI: an integrated web based platform for prediction and design of HIV proteins inhibitors
Source: J Cheminform. 2018 Mar 9;10:12. doi: 10.1186/s13321-018-0266-y (PMC5845081; doi:10.1186/s13321-018-0266-y)
Supplement: Supplementary file 2 — Additional file 2. Source code of HIVProtI web server. [file 13321_2018_266_MOESM2_ESM.zip › HIVprotI_Source-code/hivprotI/weka-3-6-6/doc/weka/core/xml/XMLSerializationMethodHandler.html]

XMLSerializationMethodHandler


---


|  |  |  |
| --- | --- | --- |
| **Overview** \| **Package** \| **Class** \| **Tree** \| **Deprecated** \| **Index** \| **Help** \| **Weka's home** | |  |
| **PREV CLASS**   **NEXT CLASS** | **FRAMES**    **NO FRAMES**     **All Classes** |  |
| SUMMARY: NESTED \| FIELD \| CONSTR \| METHOD | DETAIL: FIELD \| CONSTR \| METHOD |  |


---


## weka.core.xml Class XMLSerializationMethodHandler

```
java.lang.Object
  weka.core.xml.XMLSerializationMethodHandler
```

**All Implemented Interfaces:**: RevisionHandler

---

``` public class XMLSerializationMethodHandler extends java.lang.Object implements RevisionHandler ```

This class handles relationships between display names of properties
(or classes) and Methods that are associated with them. It differentiates
between read and write methods. It automatically stores public methods that
have the same signature as the `readFromXML()` and
`writeToXML()` methods in the `XMLSerialization`
class.

**Version:**
:   $Revision: 1.5 $

**Author:**
:   FracPete (fracpete at waikato dot ac dot nz)

**See Also:**: `MethodHandler`, `XMLSerialization`

---

**Constructor Summary**

`XMLSerializationMethodHandler(java.lang.Object owner)`
  
          initializes the method handling, executes also `clear()`, which
adds initial methods automatically.


| **Method Summary** | |
| --- | --- |
| `void` | `clear()`             removes all current methods and adds the methods according to the |
| `static java.lang.reflect.Method` | `findReadMethod(java.lang.Object o, java.lang.String name)`             returns the method with the given name that has the same signature as `readFromXML()` of the `XMLSerialiation` class. |
| `static java.lang.reflect.Method` | `findWriteMethod(java.lang.Object o, java.lang.String name)`             returns the method with the given name that has the same signature as `writeToXML()` of the `XMLSerialiation` class. |
| `java.lang.String` | `getRevision()`             Returns the revision string. |
| `MethodHandler` | `read()`             returns the handler for read methods |
| `void` | `register(java.lang.Object handler, java.lang.Class cls, java.lang.String name)`             adds read and write methods for the given class, i.e., read&;lt;name> and write<name> ("name" is prefixed by read and write) |
| `java.lang.String` | `toString()`             returns the read and write method handlers as string |
| `MethodHandler` | `write()`             returns the handler for write methods |

**Methods inherited from class java.lang.Object**

`equals, getClass, hashCode, notify, notifyAll, wait, wait, wait`

**Constructor Detail**

### XMLSerializationMethodHandler

```
public XMLSerializationMethodHandler(java.lang.Object owner)
                              throws java.lang.Exception
```

:   initializes the method handling, executes also `clear()`, which
    adds initial methods automatically.

    **Parameters:**: `owner` - the owner to retrieve the methods from **Throws:**: `java.lang.Exception` - if initialization fails **See Also:**: `clear()`


**Method Detail**

### findReadMethod

```
public static java.lang.reflect.Method findReadMethod(java.lang.Object o,
                                                      java.lang.String name)
```

:   returns the method with the given name that has the same signature as
    `readFromXML()` of the `XMLSerialiation` class.
    simplifies the adding of custom methods.

    :   **Parameters:**: `o` - the object to inspect: `name` - the name of the method to return **Returns:**: either `null` if no method was found or a reference **See Also:**: `XMLSerialization.readFromXML(Element)`

---


### findWriteMethod

```
public static java.lang.reflect.Method findWriteMethod(java.lang.Object o,
                                                       java.lang.String name)
```

:   returns the method with the given name that has the same signature as
    `writeToXML()` of the `XMLSerialiation` class.
    simplifies the adding of custom methods.

    :   **Parameters:**: `o` - the object to inspect: `name` - the name of the method to return **Returns:**: either `null` if no method was found or a reference **See Also:**: `XMLSerialization.writeToXML(Element, Object, String)`

---


### clear

```
public void clear()
```

:   removes all current methods and adds the methods according to the

---


### read

```
public MethodHandler read()
```

:   returns the handler for read methods

    :   **Returns:**: the methodhandler for read methods

---


### write

```
public MethodHandler write()
```

:   returns the handler for write methods

    :   **Returns:**: the methodhandler for read methods

---


### register

```
public void register(java.lang.Object handler,
                     java.lang.Class cls,
                     java.lang.String name)
```

:   adds read and write methods for the given class, i.e., read&;lt;name>
    and write<name> ("name" is prefixed by read and write)

    :   **Parameters:**: `handler` - the handler class that contains the read and write method: `cls` - the class to register the read and write method for: `name` - the suffix of the read and write method

---


### toString

```
public java.lang.String toString()
```

:   returns the read and write method handlers as string

    :   **Overrides:**: `toString` in class `java.lang.Object`
    :   **Returns:**: the read/write method handlers as string

---


### getRevision

```
public java.lang.String getRevision()
```

:   Returns the revision string.

    :   **Specified by:**: `getRevision` in interface `RevisionHandler`
    :   **Returns:**: the revision


---


|  |  |  |
| --- | --- | --- |
| **Overview** \| **Package** \| **Class** \| **Tree** \| **Deprecated** \| **Index** \| **Help** \| **Weka's home** | |  |
| **PREV CLASS**   **NEXT CLASS** | **FRAMES**    **NO FRAMES**     **All Classes** |  |
| SUMMARY: NESTED \| FIELD \| CONSTR \| METHOD | DETAIL: FIELD \| CONSTR \| METHOD |  |


---
